# Interferometric Fiber Optic Sensors

**DOI:** 10.3390/s120302467

**Published:** 2012-02-23

**Authors:** Byeong Ha Lee, Young Ho Kim, Kwan Seob Park, Joo Beom Eom, Myoung Jin Kim, Byung Sup Rho, Hae Young Choi

**Affiliations:** 1 School of Information and Communications, Gwangju Institute of Science and Technology, 123 Cheomdan-gwagiro, Buk-gu, Gwangju 500-712, Korea; E-Mails: kyh0789@gist.ac.kr (Y.H.K.); ganseobi@gist.ac.kr (K.S.P.); 2 Korea Photonics Technology Institute, Cheomdanbencheo-ro, Buk-gu, Gwangju 500-779, Korea; E-Mails: jbeom@kopti.re.kr (J.B.E.); mjinkim@kopti.re.kr (M.J.K.); kalparho@kopti.re.kr (B.S.R.); 3 Medical Device Development Center, Osong Medical Innovation Foundation, 186 Osong Saengmyung-ro, Gangoe-myeon, Cheongwon-gun, Chungbuk 363-951, Korea; E-Mail: chy4745@gmail.com

**Keywords:** fiber-optic sensors, fiber interferometers, Fabry-Perot interferometers, Mach-Zehnder interferometers, Michelson interferometers, Sagnac interferometers

## Abstract

Fiber optic interferometers to sense various physical parameters including temperature, strain, pressure, and refractive index have been widely investigated. They can be categorized into four types: Fabry-Perot, Mach-Zehnder, Michelson, and Sagnac. In this paper, each type of interferometric sensor is reviewed in terms of operating principles, fabrication methods, and application fields. Some specific examples of recently reported interferometeric sensor technologies are presented in detail to show their large potential in practical applications. Some of the simple to fabricate but exceedingly effective Fabry-Perot interferometers, implemented in both extrinsic and intrinsic structures, are discussed. Also, a wide variety of Mach-Zehnder and Michelson interferometric sensors based on photonic crystal fibers are introduced along with their remarkable sensing performances. Finally, the simultaneous multi-parameter sensing capability of a pair of long period fiber grating (LPG) is presented in two types of structures; one is the Mach-Zehnder interferometer formed in a double cladding fiber and the other is the highly sensitive Sagnac interferometer cascaded with an LPG pair.

## 1. Introduction

Over the last few decades optical fibers have been widely deployed in telecommunication industries owing to their special performance as the best light guidance [1]. In addition, with the development of optoelectronic technology [2], optical fibers have been intensively investigated at various sensor fields owing to their unique characteristics such as multiplexing, remote sensing, high flexibility, low propagating loss, high sensitivity, low fabrication cost, small form factor, high accuracy, simultaneous sensing ability, and immunity to electromagnetic interference. To date, a variety of studies have been made to utilize optical fibers as sensing indicators for temperature, strain, pressure, rotation, displacement, refractive index (RI), polarization, ultrasound, and so on [3–10]. Furthermore, their sensing abilities have been considerably enhanced by utilizing innovative fiber optic technologies of fiber gratings, fiber interferometers, Brillouin/Raman scattering, surface Plasmon resonance (SPR), micro-structured fibers, nano-wires, specialty fiber couplers, *etc.* [11–16]. Indeed, some fiber optic sensors are used for real time deformation monitoring of aircrafts, ships, bridges, and constructions [17]. With the development of human-friendly smart materials, even health monitoring systems using fiber devices have attracted a great interest as future technologies. In spite of their increasing research outputs and extending application areas, mainly due to the relatively high cost of optical devices, only a few of them have been commercialized. Even in the sensor market, the fiber-optic sensors represent only a small portion. However, there is no doubt about their great industrial potential for the future.

This paper aims to review and categorize fiber optic interferometric sensors according to their operating principles, fabrication methods, and application fields. Due to the immense range of sensing schemes, it is difficult to cover all types of fiber sensors in this limited review. Thus, only the fiber optic interferometric sensors are treated. A fiber optic interferometer uses the interference between two beams that have propagated through different optical paths of a single fiber or two different fibers. So, they require beam splitting and beam combining components in any configurations [16]. Of course, one of the optical paths should be arranged to be easily affected by external perturbations. Since the interferometers give a lot of temporal and spectral information as their signal, the measurand can be quantitatively determined by various means of detecting the changes in the wavelength, phase, intensity, frequency, bandwidth, and so on. With these sensing indicators, they can give remarkable performance in large dynamic range, high accuracy, and high sensitivity [1]. The current trend of fiber optic interferometers is to miniaturize them for micro-scale applications. Thus, traditional bulk optic components such as beam splitters, combiners, and objective lenses have been rapidly replaced by small-sized fiber devices that enable the sensors to operate on fiber scales. As a best candidate to implement miniaturized fiber optic interferometers, in-line structures which have two optical paths in one physical line have been widely investigated. The in-line structure offers several advantages such as easy alignment, high coupling efficiency, and high stability.

## 2. Types of Interferometric Fiber Optic Sensors

There exist representative four types of fiber optic interferometers, called the Fabry-Perot, Mach-Zehnder, Michelson, and Sagnac. For each type of sensor, the operating principles and the fabrication processes are presented. Then, some of their characteristics for sensing applications are described with some recently reported research in each field.

### 2.1. Fabry-Perot Interferometer Sensor

**Figure 1.**
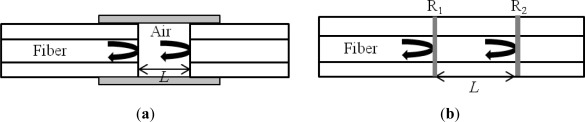
(**a**) Extrinsic FPI sensor made by forming an external air cavity, and (**b**) intrinsic FPI sensor formed by two reflecting components, R_1_ and R_2_, along a fiber.

A Fabry-Perot interferometer (FPI) is generally composed of two parallel reflecting surfaces separated by a certain distance [18]. Sometimes it is called an etalon [19]. Interference occurs due to the multiple superpositions of both reflected and transmitted beams at two parallel surfaces [20]. For the fiber optic cases, the FPI can be simply formed by intentionally building up reflectors inside or outside of fibers. FPI sensors can be largely classified into two categories: one is extrinsic and the other is intrinsic [21,22]. The extrinsic FPI sensor uses the reflections from an external cavity formed out of the interesting fiber [17]. Figure 1(a) shows an extrinsic FPI sensor, in which the air cavity is formed by a supporting structure. Since it can utilize high reflecting mirrors, the extrinsic structure is useful to obtain a high finesse interference signal [23]. Furthermore, the fabrication is relatively simple and does not need any high cost equipment. However, the extrinsic FPI sensors have disadvantages of low coupling efficiency, careful alignment, and packaging problem [19]. On the other hands, the intrinsic FPI fiber sensors have reflecting components within the fiber itself. For example, when the reflectors are formed within a fiber by any means, as in Figure 1(b), we can have the intrinsic FP interference. The local cavity of the intrinsic FPI can be formed by a lot of methods such as micro machining [24–27], fiber Bragg gratings (FBGs) [28,29], chemical etching [30,31], and thin film deposition [32,33]. However, they still have a problem of using high cost fabrication equipment for the cavity formation. In other sense, when the cavity material is not the fiber itself, it is called extrinsic. However, the definition becomes vague due to the advent of specialty fibers and fiber devices.

The reflection or transmission spectrum of an FPI can be described as the wavelength dependent intensity modulation of the input light spectrum, which is mainly caused by the optical phase difference between two reflected or transmitted beams. The maximum and the minimum peaks of the modulated spectrum mean that both beams, at that particular wavelength, are in phase and out-of-phase, respectively, in the modulus of 2π. The phase difference of the FPI is simply given as:

(1) $$\delta_{\mathrm{FPI}}=\frac{2\pi }{\lambda }n2L$$

where *λ* is the wavelength of incident light, *n* is the RI of cavity material or cavity mode, and *L* is the physical length of the cavity. When perturbation is introduced to the sensor, the phase difference is influenced with the variation in the optical path length difference (OPD) of the interferometer. Applying longitudinal strain to the FPI sensor, for instance, changes the physical length of the cavity or/and the RI of the cavity material, which results in phase variation. By measuring the shift of the wavelength spectrum of a FPI, the strain applied on it can be quantitatively obtained. The free spectral range (FSR), the spacing between adjacent interference peaks in a spectrum, is also influenced by the OPD variation. The shorter OPD gives the larger FSR. Even though a large FSR gives a wide dynamic range to a sensor, at the same time, it gives a poor resolution due to blunt peak signals [34]. Therefore, depending on applications, it is important to design the OPD of the FPI for satisfying both the dynamic range and the resolution.

**Figure 2.**
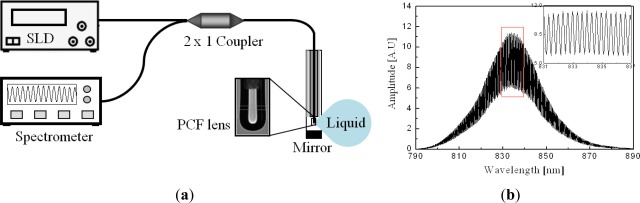
(**a**) Schematic of an extrinsic FPI liquid RI sensor system based on a PCF lens, and (**b**) its reflection spectrum measured with an air cavity [35].

For measuring the RI of liquid, extrinsic FPI sensors are appropriate because the measurand can easily access the cavity. There has been an attempt to measure the liquid RI with the cavity-accessible intrinsic structure that is formed by a micro-hole, but it required elaborate laser machining process [24,25]. Figure 2(a) illustrates the extrinsic sensor configuration based on a phonic crystal fiber (PCF) lens [35,36]. The problem of low coupling efficiency of extrinsic FPI sensors could be overcome by introducing a PCF and a fiber lens on it. By using the electric arc discharge of a conventional fusion splicer, the air holes of the PCF could be collapsed and a lens was easily formed on its distal end. The PCF lens having a properly optimized curvature effectively acted as both a beam reflector and a collimator at the same time. The spectrum of a fabricated sensor, measured with an air cavity, is shown in Figure 2(b) [35]. We can see a high contrast sinusoidal interference fringe pattern especially in the magnified inset. As applying a series of liquid solutions having RIs from 1.400 to 1.438 with a step of 0.002 into the cavity, the reflection spectra were measured and their inverse fast Fourier transforms (IFFT) were taken in order to characterize them in the Fourier frequency domain.

**Figure 3.**
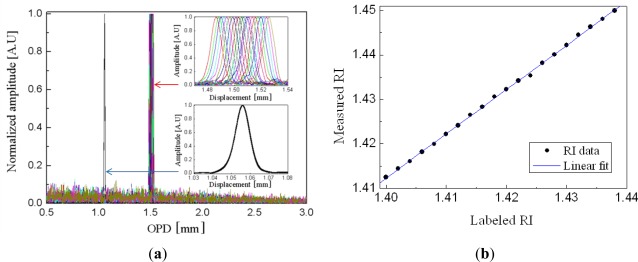
(**a**) Fourier spectra of the fabricated FPI fiber sensor measured with liquid (upper inset) and without liquid (lower inset) at the cavity, and (**b**) the RI of the liquid calculated from the Fourier spectrum and plotted with respect to the labeled RI. A series of liquid solutions of RIs from 1.400 to 1.438 with a step of 0.002 were applied into the cavity [35].

Figure 3(a) (see the lower inset) shows that the Fourier spectrum of Figure 2(b) has only one dominant peak corresponding to a half OPD of around 1 mm, the physical length of the cavity. The spectra measured with the series of liquid solutions at the cavity are presented with the upper inset of the figure. With increasing the RI, the Fourier peak is gradually shifted to the longer OPD, which means the optical length of the cavity increases with the RI of the liquid in it. In order to confirm the reproducibility of the sensor, the spectrum of the air cavity was repeatedly measured after every measurements of the liquid cavity. The lower inset of Figure 3(a) is composed of 20 Fourier spectra but looks like a single spectrum, which means the repeatability is excellent. From the Fourier peak variation, the RI of the liquid was calculated and compared with the labeled RI of the liquid as shown in Figure 3(b). We can see the data points are well fitted with a linear curve. However, since the labeled RI was measured at a wavelength of 589 nm, different from our measurement done at 835 nm, there exists a constant offset between two RI data [35]. Measurement in the Fourier domain has the limitation in resolving the Fourier peaks when there are several peaks nearby. Generally, the spatial resolution in the Fourier domain is defined by the bandwidth of a single resolved Fourier peak, which is determined by the spectral bandwidth of a light source. In our experiment, the source bandwidth was about 50 nm and the spatial bandwidth was about 10 μm. However, the FPI sensor based on a PCF lens has only one Fourier peak, which means only one cavity mode is involved, so that the Fourier peak overlap does not occur except near the DC peak. In this case, the accuracy or sensitivity of the sensor are important and given by how accurately the position of a Fourier peak position can be read. Of course, the spectral resolution of the spectrometer is a key factor, but disturbance of the source power and system instability affect the accuracy also. With a cavity length of 1 mm and a spectrometer resolution of 0.05 nm, the RI resolution of 2.6 × 10^−5^ was calculated [35]. As a result, this extrinsic FPI fiber sensor based on a specialty fiber PCF and a PCF lens is simple but very accurate, so that it should be useful for real time RI measurements of various liquid samples including gasoline, alcohol, and polluted water.

The extrinsic structure has the merit of sensing displacement since the phase value of FPI signal can be directly affected by the displacement of the external reflecting surface [4]. By applying polymer thin films as the reflecting surfaces, furthermore, pressure and ultrasound sensors have been widely implemented with the extrinsic FPI configuration [9,33]. Because the Young’s modulus of polymer is much lower than that of fiber material, the polymer film can be used as a deformable cavity for the measurement of pressure or ultrasound.

**Figure 4.**
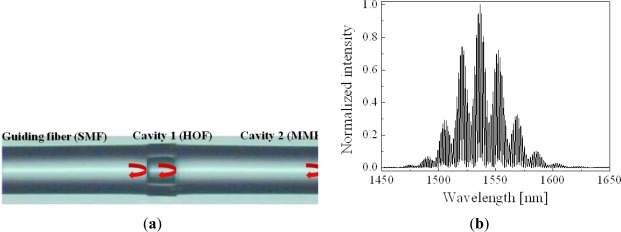
(**a**) The microscope image of an implemented double cavity FPI fiber sensor, and (**b**) its reflection spectrum [38]. The length of HOF is ∼70 μm, and the length of MMF is ∼360 μm. SMF; single mode fiber, HOF; hollow optical fiber, MMF; multi mode fiber.

**Figure 5.**
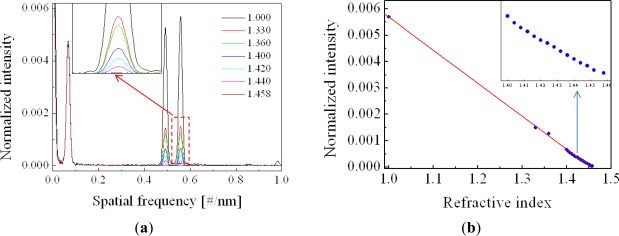
(**a**) Fourier spectra measured with air (black curve) and several RI solutions (color curve). Inset is the magnified image of the red dotted region; (**b**) Intensity variation of the third Fourier peak plotted with respect to the labeled RI of the solutions [38].

In addition to the extrinsic FPI sensors, a variety of intrinsic FPI sensors have been developed with various fiber structures. Among them, a double cavity structure fiber FPI is unique and interesting [37–39]. As shown in Figure 4(a), the double cavity can be simply formed by fusion-splicing a short piece of holey optical fiber (HOF) between a single mode fiber (SMF) and a piece of multimode fiber (MMF) [38]. The reflection spectrum of the double cavity FPI sensor, implemented with a piece of HOF of a length of ∼70 μm and a MMF of ∼360 μm, is shown in Figure 4(b). It has a rather complex fringe pattern due to the superposition of two cavities. The sinusoidal interference fringe is fast oscillating within a slowly varying envelop curve. However, the Fourier spectrum, Figure 5(a), shows that there are three dominant peaks except the DC peak resulting from the light source spectrum. The first peak near DC is caused by the short cavity 1 and the second peak is by the rather long cavity 2. The third peak is due to the combination of both cavities. When the distal end of the MMF was put into a liquid solution, the intensities of the second and the third peaks were decreased but the first peak was not changed as shown in Figure 5(a). It is understood that the Fresnel reflection only at the MMF end surface decreases with the RI of the liquid solution. The RI of the liquid solution calculated from the intensity variation of the Fourier peak is plotted in Figure 5(b) [38]. In this case, though the FPI uses air cavity, not a fiber cavity, since the HOF itself is a kind of fiber, it can be categorized as an intrinsic fiber sensor.

The double cavity fiber FPI can be used as a temperature sensor also. Since the HOF cavity and the MMF cavity have different thermo-optic coefficients, the temperature-induced movement of the first peak is different from the movements of the other peaks [37]. By coating a gas sensitive material on the end face of the MMF cavity, it can be also utilized as a gas sensor. With coating palladium, which is a hydrogen-sensitive metal, a hydrogen gas sensor could be implemented [39]. By adjusting the lengths of multiple MMF cavities, multiplexing several sensors is also possible. With the similar configuration, but chemically etching the cores of SMFs and fusion-splicing them in series, a multi-cavity FPI biosensor was implemented [30]. One thing to emphasize as a beneficial point of this kind of double cavity FPI fiber sensors is that the inner cavity is not affected by the variation of the outer chemical and physical environments. Therefore, the fatal measurement error of the intensity-based sensor, such as source power fluctuation or external disturbance, can be compensated by using the first Fourier peak [39].

Intrinsic cavities have been implemented by many types of specialty optical fiber devices such as micro-structured optical fibers (MOFs) or FBGs [40–43]. The environmental stability of MOFs is better than that of conventional SMFs. Especially the strain sensing ability at specific external disturbing conditions could be successfully demonstrated with MOFs [41,42]. Of course, as a wavelength-dependent distributed reflector, FBG has played a great role in making a high resolution and multiplexing sensor [29,43]. It is sure that the application fields of FPI sensors would be extended with the development of specialty optical fiber and related devices.

### 2.2. Mach-Zehnder Interferometer Sensors

**Figure 6.**
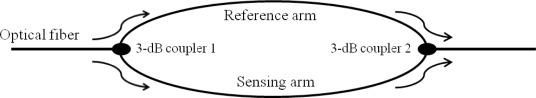
The schematic of an MZI. A beam is split into two arms, the reference and the sensing arms, and then recombined by using two fiber couplers.

Mach-Zehnder interferometers (MZIs) have been commonly used in diverse sensing applications because of their flexible configurations. Early MZIs had two independent arms, which are the reference arm and the sensing arm, as illustrated in Figure 6. An incident light is split into two arms by a fiber coupler and then recombined by another fiber coupler. The recombined light has the interference component according to the OPD between the two arms. For sensing applications, the reference arm is kept isolated from external variation and only the sensing arm is exposed to the variation. Then, the variation in the sensing arm induced by such as temperature, strain, and RI changes the OPD of the MZI, which can be easily detected by analyzing the variation in the interference signal.

**Figure 7.**
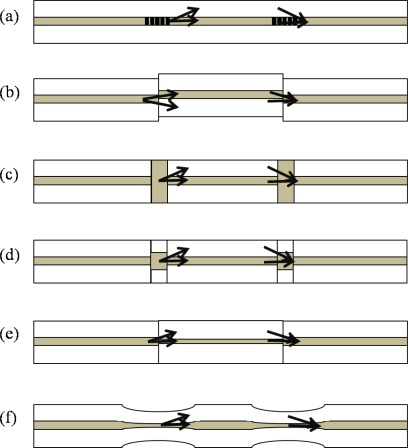
Configuration of various types of MZIs; the methods of using (**a**) a pair of LPGs, (**b**) core mismatch, (**c**) air-hole collapsing of PCF, (**d**) MMF segment, (**e**) small core SMF, and (**f**) fiber tapering.

The scheme of using two separated arms in the MZIs has been rapidly replaced with the scheme of in-line waveguide interferometer since the advent of long period fiber gratings (LPGs). As shown in Figure 7(a), a part of the beam guided as the core mode of a SMF is coupled to cladding modes of the same fiber by an LPG, and then re-coupled to the core mode by another LPG.

The combined beam and the uncoupled beam in the core make interference, which gives a compact but very effective MZI. This in-line type of MZIs has the same physical lengths in both the reference arm and the sensing arm, but has the different optical path lengths due to the modal dispersion; the cladding mode beam has a lower effective index than the core mode beam. The LPGs are generally fabricated by inducing periodic modulation in the RI of the fiber core by UV light, CO_2_ laser, or mechanical press [44–49]. Even with the photonic crystal fiber (PCFs) made of a single material, pure silica, a pair of LPGs could be made by applying periodic pressure along the PCF [44].

An MZI temperature sensor using LPGs has been presented [45], where the thermo-optic coefficient of the fiber core material was analyzed by using a pair of LPGs. The fine interference fringe enabled to calculate even the wavelength dependency of the effective index of the core mode. It also showed that the Germanium-doped core had a stronger thermo-optic coefficient than the Boron co-doped core. An RI sensor based on the MZI composed of a pair of LPGs has been also reported [46], where the sensitivity of the cladding mode to the RI change in the surrounding medium was utilized. A sensitivity as fine as 1.8 × 10^−6^ was achieved. In order to improve the sensitivity to RI, the method of fiber tapering has been applied to the separated region between two LPGs [49]. However, the LPG pair MZI has a problem in the operating wavelength. The LPG is working only in a limited band(s) of wavelengths due to the phase matching phenomenon of fiber gratings. Further, both LPGs should be identical to get the maximum performance [50].

Another way of splitting a beam into the core and the cladding modes of a fiber is splicing two fibers with a minute lateral offset as shown in Figure 7(b). Due to the offset, a part of the core mode beam is coupled to several cladding modes without being heavily affected by the wavelength. Even with PCF, an MZI can be formed by simply fusion-splicing a piece of PCF between fibers with a small intentional deviation [51]. The offset method is cost effective and fast in comparison with the LPG pair method. Also, we can use any wavelength for operation. Of course, the number of involved cladding modes and the insertion loss can be controlled by adjusting the amount of offset. In reference [51], as low as 2 dB splicing loss was achieved by making the mode coupling to dominantly one cladding mode of the PCF. Collapsing air holes of a PCF is another good way of making an in-line MZI. It is easy and does not need any troublesome cleaving or aligning process. The core mode beam in a PCF is expanded at the air hole collapsed region, so that a part of it could be coupled to the cladding modes of the PCF, as shown in Figure 7(c). However, in this case, coupling to several cladding modes was observed and controlling the number of involved modes was not so simple [51]. Further, the insertion loss was rather high compared with the offset method. By combining the LPG method and the collapsing method, the insertion loss could be reduced by ∼3 dB [52]. These PCF-based in-line MZI sensors have several advantages including operation in high temperatures and low cross sensitivity owing to not using doped cores compared with conventional SMFs. However, most of the fiber optic in-line MZIs are based on multimode interference. The cladding part of a SMF is a multimode waveguide, so that the number of cladding modes involving the MZI is more than one in general. Exceptionally, the LPG pair uses only one cladding mode in most cases [47]. Such multimode interference affects the sensing performance because each mode has a different sensitivity to the external variations. Therefore, it is necessary to minimize the number of involving cladding modes in sensor fabrication and also the analysis should be made with considering the multimode interference carefully.

Another method for splitting the beam in a fiber is to use the fibers having different core sizes as shown in Figure 7(d,e) [53,54]. Figure 7(d) shows one method in which a short piece of MMF is fusion spliced into a SMF at two points along the SMF. In this case, the light propagating through the core of the SMF is spread at the MMF region and then coupled into the core and cladding of the next SMF [53]. Figure 7(e) shows another method, in which a small core fiber is inserted between two conventional SMFs. At the small core fiber region, the beam is guided not only as the core mode but also as the cladding mode [54]. By tapering a fiber at two points along the fiber, we can form an effective in-line MZI as shown in Figure 7(f) [55,56]. Due to the tapering, the core mode diameter is increased so that a part of it could be coupled to cladding mode(s). It is cost effective and relatively very simple but mechanically weak especially at the tapering region. Beside theses, there have been MZIs using a double cladding fiber [57], micro-cavities [58], and a twin-core fiber [59].

**Figure 8.**
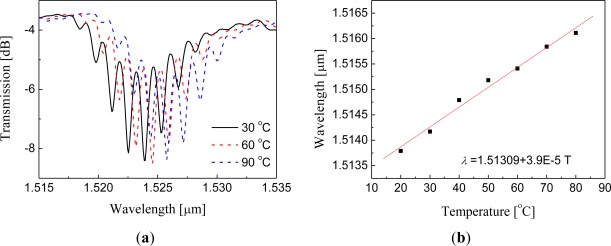

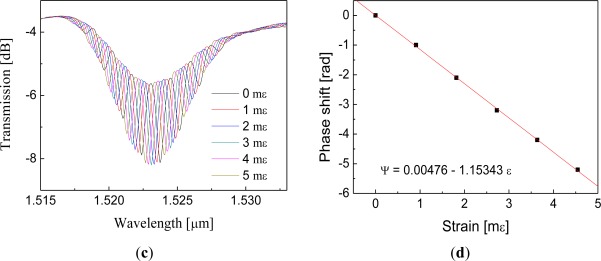
(**a**) Transmission spectra of an LPG pair under temperature variations; the spectrum was shifted with the same phase; (**b**) The amount of the spectrum shift with temperature; it was shifted toward longer wavelength with the sensitivity of ∼39 pm/°C; (**c**) The spectra under strain variations; only the phase was changed without affecting the envelop curve; (**d**) The amount of the phase shift measured with strain [48].

Simultaneous measurements of several measurands are possible with the in-line MZI. By using the LPG pair made in double cladding fiber (DCF), we can simultaneously measure strain and temperature with minimizing the cross talk between them [48]. An LPG couples the core mode to several cladding modes of a fiber and this cladding modes are normally guided by the total internal reflection (TIR) at the cladding surface. In a conventional SMF, the fiber is jacketed with a resin material having a RI higher than the RI of the cladding material. Therefore, cladding modes are absorbed there, which prohibits the formation of an MZI with the coated jacket. However, for the DCF case, the cladding mode is guided by the inner cladding boundary, so that it can propagate regardless of the existence of any jacket material. Because of this property of the DCF, the grating-free region between two gratings of an LPG pair can be insensitive to physical contacts; moreover, it is possible to hold the grating-free region to isolate strain from thermal effect. Based on this, the simultaneous sensor could be made by applying strain only at the grating-free region of an LPG pair, while temperature was applied to the whole region of the LPG pair. With this configuration, the phase and the envelope of the MZI interference fringes were thermally shifted with the same rate, but the strain shifted only the phase as shown with Figure 8. Therefore, we were able to measure temperature and strain simultaneously.

### 2.3. Michelson Interferometer Sensors

**Figure 9.**
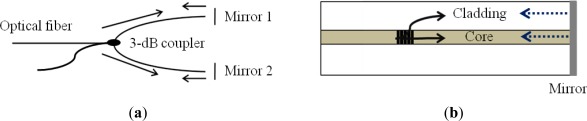
(**a**) Basic configuration of a Michelson interferometer and (**b**) schematic of a compact in-line Michelson interferometer.

Fiber-optic sensors based on Michelson interferometers (MIs) are quite similar to MZIs. The basic concept is the interference between the beams in two arms, but each beam is reflected at the end of each arm in an MI as shown in Figure 9(a) [60–63]. In fact, an MI is like a half of an MZI in configuration. Thus, the fabrication method and the operation principle of MIs are almost the same as MZIs. The main difference is the existence of a reflector(s). Since MIs use reflection modes, they are compact and handy in practical uses and installation. Multiplexing capability with parallel connection of several sensors is another beneficial point of MIs. However, it is essential to adjust the fiber length difference between the reference arm and the sensing arm of an MI within the coherence length of the light source. An in-line configuration of MI is also possible as shown with Figure 9(b). A part of the core mode beam is coupled to the cladding mode(s), which is reflected along with the uncoupled core mode beam by the common reflector at the end of the fiber.

There have been many fiber-optic sensors based on in-line MIs [64–68], especially for measurements of temperature and RIs of liquid specimens. The RI sensor based on a single LPG is easy to use practically in comparison with the MZI type LPG pair sensor, since only the grating-free region can be immersed in the liquid specimen. When even the LPG(s) is immersed in liquid, the entire LPG spectrum is shifted, which gives a cross sensitivity. This drawback of LPG-based MIs was overcome by coating the LPG with metal, which prevented the exposure to liquid [64]. A core-offset method for the RI sensing was investigated also with the MI configuration; minimum insertion loss of 0.01 dB and extinction ratio over 9 dB were obtained, which showed a comparable RI sensitivity to that of an LPG-based RI sensor [65].

**Figure 10.**
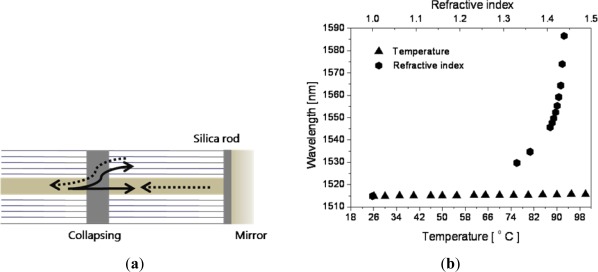
(**a**) Schematic of the PCF-based Michelson interferometer designed for temperature-insensitive liquid RI measurement, and (**b**) its spectral responses to RI and temperature [63]. A part of the core mode beam can be coupled to the cladding mode(s) by collapsing the PCF in a short length. Both beams reflected by the common mirror make interference. The silica rod fusion-spliced at the end of the PCF blocks the liquid.

Typically, fiber optic sensors have cross sensitivity among measurands; especially they are easily affected by temperature variation. By using two types of fibers having different temperature sensitivities, the temperature dependency in RI measurement could be eliminated or reduced [66]. Another approach of reducing the temperature dependency was using PCF. As mentioned earlier, the PCF made of a fused silica is almost insensitive to temperature compared with conventional core-doped fibers. However, since the PCF has many air holes, in general, the MI type PCF sensor could not be immersed in the liquid specimen. Any liquid within the air holes of a PCF reduces the wave-guiding capability of the PCF. This problem was overcome simply by splicing a short piece of silica rod at the end of the PCF, which blocks liquid going inside the holes of the PCF as illustrated in Figure 10(a) [67]. Figure 10(b) shows the PCF-based MI sensor responding simultaneously to RI and surrounding temperature. There was no appreciable wavelength shift in the interference spectrum with the temperature variation; but it was heavily shifted with the RI variations. With the liquid having an RI near to the fiber material index, more precisely near to the modal index of the corresponding cladding mode, as much as 70 nm wavelength shift was observed.

There has been another MI application for measuring flow velocity [68]. To make the MI configuration appropriate to a flow velocity sensor, tapering method was used for splitting a beam into two cores of a twin-core fiber. This sensor measured the OPD change between beams in the two cores, which was affected by the bending of the twin-core fiber induced by the flow.

### 2.4. Sagnac Interferometer Sensor

**Figure 11.**
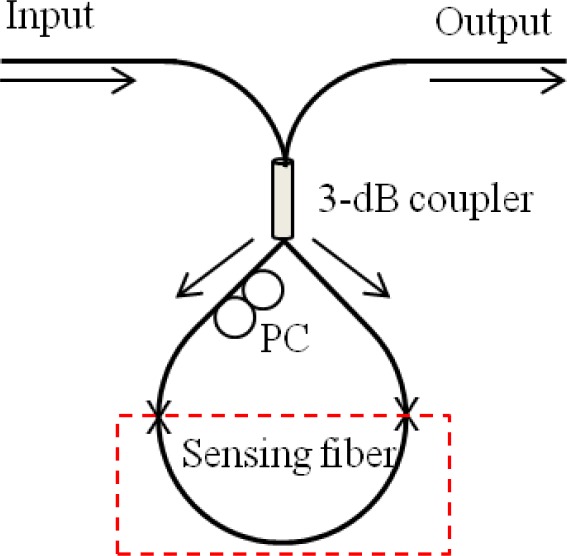
Schematic of the sensor based on a Sagnac interferometer.

Sagnac interferometers (SIs) are recently in great interest in various sensing applications owing to their advantages of simple structure, easy fabrication, and environmental robustness [69]. An SI consists of an optical fiber loop, along which two beams are propagating in counter directions with different polarization states. As schematically illustrated in Figure 11, the input light is split into two directions by a 3 dB fiber coupler and the two counter-propagating beams are combined again at the same coupler. Unlike other fiber optic interferometers, the OPD is determined by the polarization dependent propagating speed of the mode guided along the loop. To maximize the polarization-dependent feature of SIs, birefringent fibers are typically utilized in sensing parts. The polarizations are adjusted by a polarization controller (PC) attached at the beginning of the sensing fiber. The signal at the output port of the fiber coupler is governed by the interference between the beams polarized along the slow axis and the fast axis. The phase of the interference is simply given as:

(2) $$\delta_{\mathrm{SI}}=\frac{2\pi }{\lambda }\mathrm{BL},B=\left|n_{f}-n_{s}\right|$$

where *B* is the birefringent coefficient of the sensing fiber, *L* is the length of the sensing fiber, and *n_f_* and *n_s_* are the effective indices of the fast and slow modes, respectively [69].

In general, high birefringent fibers (HBFs) or polarization maintaining fibers (PMFs) are chosen as the sensing fibers to acquire a high phase sensitivity. For the temperature sensing application, the fiber is doped to have a large thermal expansion coefficient, which induces high birefringence variation [70]. When measuring others parameters such as strain, pressure, and twist, however, the high birefringent characteristics of the HBFs and PMFs can depreciate the sensing ability due to their strong temperature dependency [71–77]. In order to overcome this problem, polarization-maintaining photonic crystal fibers (PMPCFs) have been introduced as the sensing fibers. The pure silica-based PCF is good to have thermal robustness; but the air-hole structure of the PCF should be adjusted to have high birefringence [75–78]. Furthermore, since the polarization modes of the fiber are sensitive to the bending of the fiber due to the asymmetric fiber structure, a high-sensitive curvature sensor could be reported based on a simple SI setup [79].

**Figure 12.**
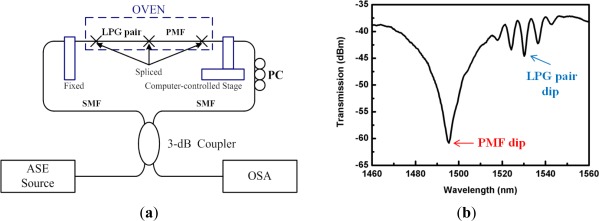
(**a**) Experimental setup of the Sagnac interferometric sensor combined with an LPG pair MZI sensor, and (**b**) the measured transmission spectrum [85]. It can measure temperature and strain simultaneously.

**Figure 13.**
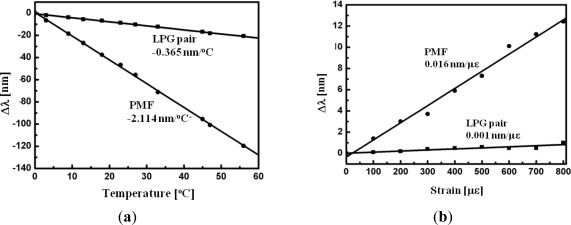
(**a**) Temperature sensitivity of the hybrid Sagnac-MZI sensor, and (**b**) its strain sensitivity. From their different sensitivities, the temperature and the strain applied on the sensor could be separated [85].

Another beneficial point of the SI-based sensors is the simultaneous sensing capability with the help of other fiber optic devices [80–85]. Figure 12(a) shows the experimental setup of hybrid Sagnac-MZI sensor based on a PMF and an LPG pair for measuring temperature and strain simultaneously [85]. The fiber loop was composed by cascading a piece of PMF and a pair of LPGs within a fiber loop. As illustrated in Figure 12(a), the sensing fiber was fixed at a computer-controlled stage for the strain measurement and also put into an oven for the temperature measurement. The measured transmission spectrum is presented in Figure 12(b) [85]. We can see two interference signals within the interesting wavelength range. The left big peak around 1500 nm is caused by the PMF SI and the other fine one around 1530 nm is formed by the LPG pair MZI. Figure 13(a) shows the temperature response of the hybrid Sagnac-MZI sensor. As increasing the temperature, both interference signals were shifted to the shorter wavelength direction; however, the sensitivity of the PMF was 4 times higher than that of the LPG pair. The strain response of the sensor is shown in Figure 13(b); the PMF was more sensitive to the strain compared to the LPG pair. From the different sensitivities of the PMF SI and the LPG pair MZI, temperature and strain could be measured simultaneously with the same fiber loop.

## 3. Conclusions

We have presented an overview on interferometric fiber-optic sensors and their applications. We first provided a brief explanation on the Fabry-Perot interferometers (FPIs) and their working principle. The extrinsic FPI liquid index sensor that had a simple structure but improved coupling efficiency, implemented by using photonic crystal fiber (PCF) and PCF lens, was highlighted. Also, the double cavity intrinsic FPI sensor, implemented by only fusion-splicing different fibers in series, was introduced for measuring refractive index (RI). Secondly, a number of fabrication methods and applications of Mach-Zehnder interferometers (MZIs) and Michelson interferometers (MIs) were briefly summarized. Their fabrication processes could be simplified by utilizing PCFs, which made the sensors cost-effective and robust to temperature. Thus, several methods for fabricating PCF-based interferometers were introduced and discussed. As another example of MZI sensor for measuring temperature and strain simultaneously, the cross-talk free double cladding fiber MZI sensor, based on an LPG pair, was introduced. Finally, the recent trend of Sagnac interferometers (SIs) was investigated. Polarization-dependent characteristic of SI type sensors could allow them highly sensitive to the external perturbations compared with other fiber-optic sensors. Simultaneous sensing ability of temperature and strain was demonstrated with the hybrid Sagnac-MZI sensor.

As presented, it is obvious that interferometric fiber optic sensors have great potential in practical applications such as real time deformation monitoring of aircrafts, ships, bridges, and constructions. Environmental sensors including explosive hydrogen sensor and biomedical sensors for health monitoring sensor systems are emerging fields. These novel sensors will evolve and expand their applications with the developments in specialty fibers and special fiber devices.
